# Laparoscopic repair of high rectovaginal fistula: Is it technically feasible?

**DOI:** 10.1186/1471-2482-5-20

**Published:** 2005-10-12

**Authors:** Saravanan S Kumaran, Chinnusamy Palanivelu, Alfie J Kavalakat, Ramakrishnan Parthasarathi, Murugayyan Neelayathatchi

**Affiliations:** 1 Department of Gastrointestinal Surgery, Gem Hospital, Coimbatore, India; 2 Department of Advanced Laparoscopic and Gastrointestinal Surgery, Gem Hospital, Coimbatore, India; 3 Department of Advanced Laparoscopic Surgery, Gem Hospital, Coimbatore, India; 4 Department of Endogynec Surgery, Gem Hospital, Coimbatore, India

## Abstract

**Background:**

Rectovaginal fistula (RVF) is an epithelium-lined communication between the rectum and vagina. Most RVFs are acquired, the most common cause being obstetric trauma. Most of the high RVFs are repaired by conventional open surgery. Laparoscopic repair of RVF is rare and so far only one report is available in the literature.

**Methods:**

We present a case of high RVF repaired by laparoscopy. 56-year-old female who had a high RVF following laparoscopic assisted vaginal hysterectomy was successfully operated laparoscopically. Here we describe the operative technique and briefly review the literature.

**Results:**

The postoperative period of the patient was uneventful and after a follow up of 6 months no recurrence was found.

**Conclusion:**

Laparoscopic repair of high RVF is feasible in selected patients but would require proper identification of tissue planes and good laparoscopic suturing technique.

## Background

Rectovaginal fistula (RVF) is an epithelium-lined communication between the rectum and vagina. Most RVFs are acquired, although congenital abnormalities do exist. Acquired RVFs can occur due to various causes. The most common acquired cause is obstetric. RVFs can be classified into low and high variety. Although perineal approach is the preferred one for low variety, high fistulas are best approached transabdominally. High RVFs are most commonly approached by a conventional open technique. We describe laparoscopic technique of repairing a case of high RVF. So far there is only one report in literature mentioning primary closure of RVF by laparoscopic technique [1].

## Methods

We are describing our technique of laparoscopic management of high RVF in a 56 year old lady who developed RVF following laparoscopic assisted vaginal hysterectomy. She was admitted with complaints of passing flatus and feces per vagina for 11 months. Laparoscopic assisted vaginal hysterectomy with bilateral salphingo-oopherectomy was done 18 months back for leiomyoma uterus. She started passing flatus and feces per vagina 4 weeks after the surgery. Clinically the patient was obese (BMI-32). Patient had no features of sphincter disturbance. Per vaginal examination revealed a small area of induration high in the vault. The vault was healthy. Routine blood investigations were normal. USG abdomen was normal. Instillation of methylene blue into the rectum with a vaginal tampon confirmed the fistula. After thorough bowel preparation, DVT prophylaxis and prophylactic antibiotics patient was taken up for surgery.

**Figure 1:**
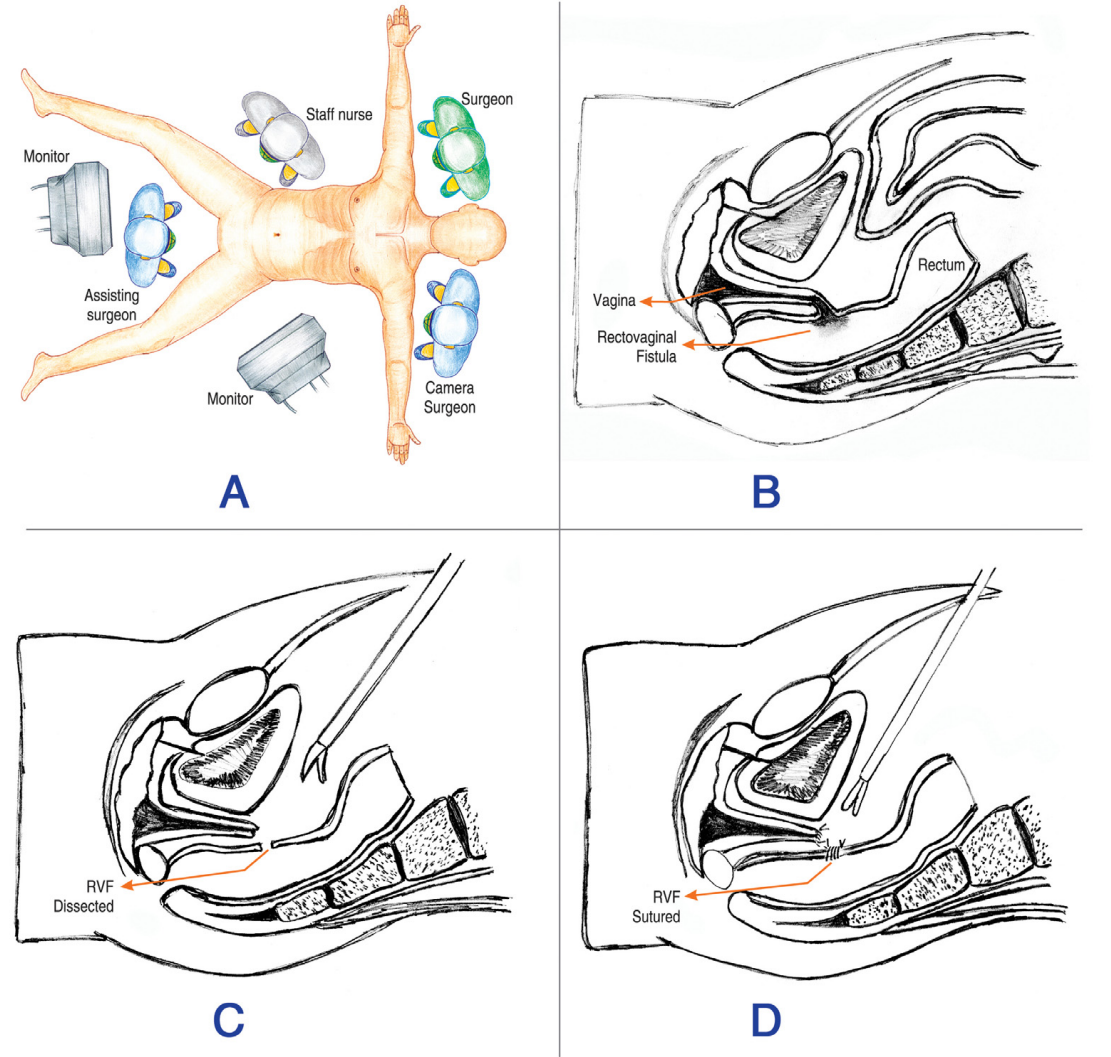
**Schematic picture of the surgical procedure**. A – Team positions. B – Picture showing RVF. C – RVF Dissected. D – Rectal and vaginal defects sutured separately.

**Figure 2:**
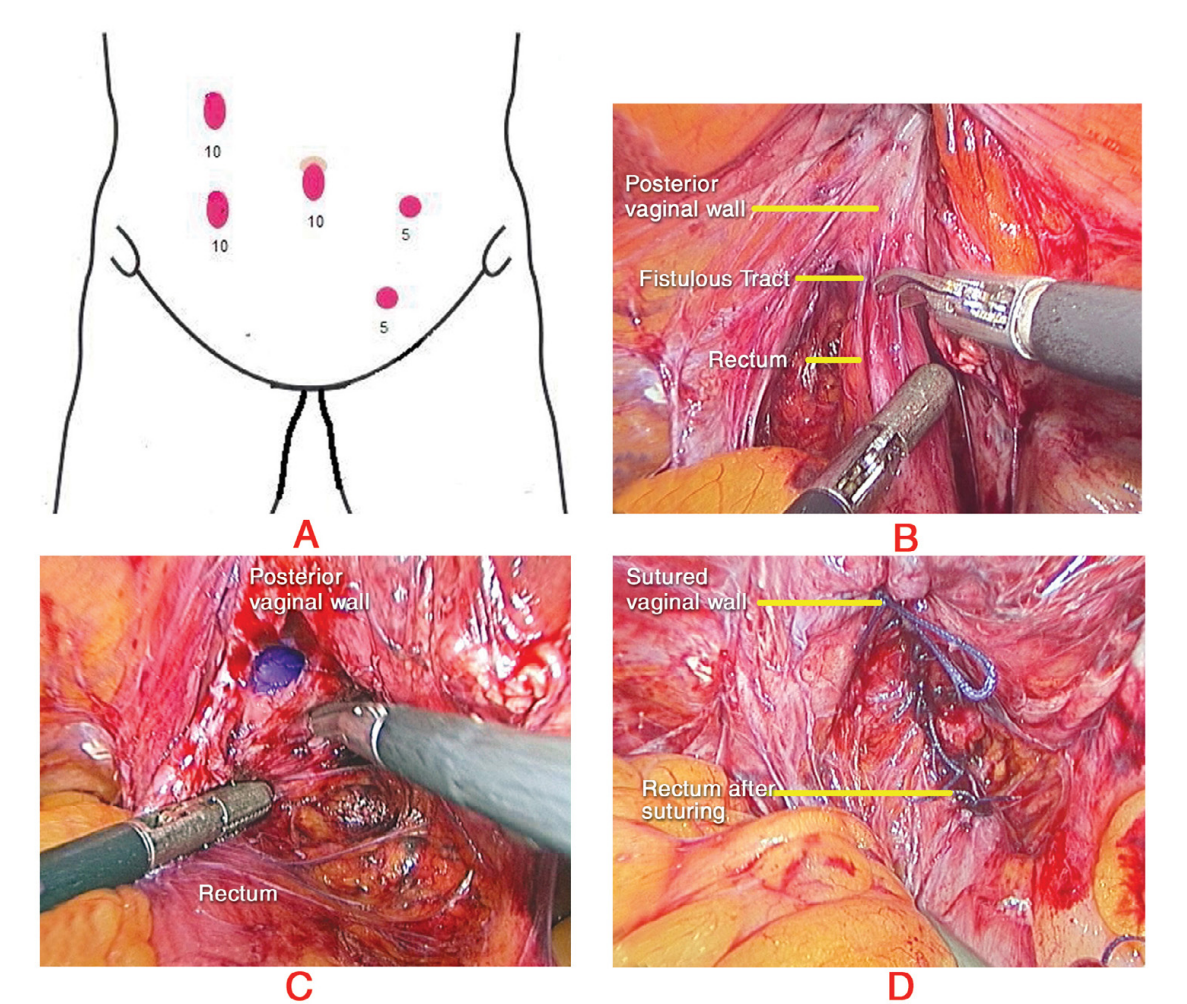
**Port Positions and Intraoperative pictures**. A – Port positions. B – Fistulous tract exposed. C – Fistulous opening on the vaginal side. D – Completion of repair.

The patient was placed in modified lithotomy position. The abdomen was prepared and draped. Cleansing of the vaginal lumen with an antiseptic solution (Povidone-iodine) was done. A Foley catheter was inserted into the urinary bladder. The surgery was performed under general anesthesia. Team position is diagrammatically represented in Figure 1A. Pneumoperitoneum was created with Veress needle. 10 mm ports were placed at the umbilicus for camera; in the right lower midclavicular line for right working hand and in the right upper midclavicular line for rectal retraction. 5 mm ports were placed in the left lower midclavicular line for left working hand and in the left iliac fossa for bladder retraction (Figure 2A).

There were adhesions between the rectum and the bladder peritoneum which was dissected using scissors. On retraction of the rectum, the dense fibrosis in the region of the fistula was identified which was divided with sharp scissors (Figure 2B). There was a 5 mm fistula between the vault of the vagina and the middle third rectum (Figure 2C). The fistulous tract was opened and thorough visualization of the fistula on the rectal and vaginal side was done. Resection of the fibrous fistulous tract was done. Mobilization of the rectum 2 cms distal to the fistula was also done.

The rent on the rectal side was closed in 2 layers with 2-0 Vicryl by intracorporeal suturing. The vaginal side of the rent was also approximated with 2-0 Vicryl in single continuous layer (Figure 2D). An omental patch was placed and sutured in situ between the repaired rectum and vagina. Thorough wash was given. Drain was placed in the pelvis through the right flank. Proximal colostomy was not performed.

## Results

The postoperative period was uneventful. The patient was started on oral fluids on the 3^rd ^postoperative day. The drain was removed on 5^th ^postoperative day and discharged on the same day. On follow up (6 Months), the patient had no specific complaints. There was no recurrence which was confirmed by performing vaginography.

## Discussion

Most RVFs are acquired. Acquired fistulas can occur due to various causes. These include trauma (including operative, obstetric, and traumatic injuries), infection, inflammatory bowel disease (IBD), carcinoma and radiation. The vaginal passage of gas and stool can cause physical symptoms due to inflammation and irritation. Patients may also suffer from significant psychosocial and sexual dysfunction.

In general, obstetric trauma is the most common cause of RVFs, occurring in up to 88% of published series [2-5]. But this is not the case at some institutions due to their referral pattern. For example, in a series from the Mayo Clinic, only 11% of their rectovaginal fistulas were secondary to obstetric injuries whereas 24% were due to inflammatory bowel disease [6]. Surgical trauma is another etiology for a RVF. Both anorectal and vaginal operations present a risk. Abdominal surgeries like hysterectomies, low anterior resections and ileo-anal anastomosis also carries the risk of developing a RVF. The fistula may result from a direct injury during the surgery or from infection or anastomotic leak postoperatively.

RVFs can be classified into low and high varieties. Low RVF is between the lower third of the rectum and the lower half of the vagina. A high fistula is between the middle third of the rectum and the posterior vaginal fornix. Small-sized fistulas are less than 0.5 cm in diameter, medium-sized fistulas are 0.5–2.5 cms, and large-sized fistulas exceed 2.5 cms.

A thorough history and physical examination are necessary to help identify the etiology of the fistula, as well as to assess its location, any ongoing inflammation, and whether a sphincter defect is present or not. Continence evaluation is of paramount importance in RVFs following perineal lacerations. All this information is essential prior to surgical treatment. Procto-sigmoidoscopy should be done. The fistulous opening may be seen as a small dimple or pit and occasionally can be gently probed for confirmation. Flexible endoscopy (sigmoidoscopy or colonoscopy) is used to fully evaluate the possibility of IBD. Evaluation of established RVFs with endo-rectal ultrasound and trans-vaginal ultrasound examination is more important if the patient complains of incontinence or if the underlying cause is obstetric trauma. Yee LF et al found that non-contrast endoanal ultrasound was not useful in imaging RVF and did not recommend this as a diagnostic or screening tool for the identification of a RVF [7]. High fistulas may not be readily apparent on physical examination or vaginal inspection and may even be missed by endoscopy. Methylene blue enema with a vaginal tampon in place, looking for staining on the tampon is used to confirm the diagnosis. Vaginography with a water soluble contrast medium has a reported sensitivity of 79% to 100% [8-10]. CT and MRI also play a role in the diagnosis and evaluation of the RVF as they may give insight into the underlying cause of the fistula.

The management of RVF depends on size, location, cause, anal sphincter function and overall health status of the patient. It also depends on the skill and judgment of the surgeon. Treatment of established RVFs should be surgical. The treatment of RVF must be tailored to the individual fistula. Transabdominal approach is the standard surgical approach for high fistulas. Different surgical techniques have been described. These include fistula division and closure with or without bowel resection and use of local flaps, such as the bulbocavernosus flap and a variety of muscle and musculocutaneous flaps for repair of large defects. Diversion colostomy is preferred to safeguard the anastomosis.

Perineal approach is the choice in the management of low RVFs. These include transvaginal, transperineal, transanal or conversion of the fistula into a complete perineal laceration with subsequent repair. The transanal endorectal advancement flap is the most popular technique of repairing simple low RVFs by colorectal surgeons while the gynecologists prefer the transvaginal approach. The perineal approach is not preferable because of the damage to the perineal body but the exposure is excellent and is indicated in failed cases following transanal and transvaginal approach.

Laparoscopic management of the RVFs is still in its infancy. Some surgeons have performed laparoscopic assisted procedures. Schwenk W et al reported a case of RVF for which they had performed a laparoscopic resection of the sigmoid colon with the fistulous tract and intracorporeal colorectal anastomosis [11]. Pelosi et al performed laparoscopic upper rectovaginal mobilization to facilitate the transvaginal repair of recurrent RVF [12]. Total laparoscopic repair is still rare, because of the complexity of the procedure. Nezhat CH reports correction of two cases of RVF by laparoscopy [1]. Our case is probably the first reported case of RVF following laparoscopic assisted vaginal hysterectomy, which has been repaired by a total laparoscopic technique.

We feel that simple high RVF can be repaired laparoscopically after proper assessment of the patient and the fistula. As in our case, there was no need for a diversion colostomy in cases where the fistulous tract is completely excised and a proper suturing of the defect is done. Good preparation of the bowel is essential to avoid any fecal contamination of the operative area. Adequate laparoscopic experience with proper identification of the tissue planes and the fistulous tract and meticulous surgical technique is required to accomplish a total laparoscopic repair. Laparoscopic repair of RVF has all the advantages of a minimal access surgery. It is associated with minimal wound complications, less postoperative pain and early recovery.

## Conclusion

Laparoscopic repair of RVFs can be demanding and would require proper identification of tissue planes and adequate experience in advanced laparoscopic procedures. We conclude that laparoscopic resection of simple high RVFs with primary intracorporeal closure is feasible and should be considered in selected cases as an alternative to open surgery. Safety and long term results of laparoscopic repair of RVF need to be confirmed by further studies.

## Abbreviations

RVF: Rectovaginal Fistula

IBD: Inflammatory bowel disease

BMI: Body mass index

DVT: Deep vein thrombosis

CT: Computerized tomography

MRI: Magnetic resonance imaging

## Competing interests

The author(s) declare that they have no competing interests.

## Authors' contributions

SSK: Acquisition of data and preparing the manuscript.

CP: Management of the case, critical evaluation and overall supervision.

AJK: Preparing the manuscript and critical evaluation.

RP: Collection of the data and drafting the manuscript.

MN: Management of the case and final approval

## Pre-publication history

The pre-publication history for this paper can be accessed here:


